# Common germline variation at the *TERT* locus contributes to familial clustering of myeloproliferative neoplasms

**DOI:** 10.1002/ajh.23842

**Published:** 2014-09-26

**Authors:** Roland Jäger, Ashot S Harutyunyan, Elisa Rumi, Daniela Pietra, Tiina Berg, Damla Olcaydu, Richard S Houlston, Mario Cazzola, Robert Kralovics

**Affiliations:** 1 CeMM Research Center for Molecular Medicine of the Austrian Academy of SciencesVienna, Austria; 2 Department of Hematology Oncology, Fondazione Istituto di Ricovero e Cura a Carattere Scientifico (IRCCS) Policlinico San MatteoPavia, Italy; 3 Department of Molecular Medicine, University of PaviaPavia, Italy; 4 Division of Genetics and Epidemiology, Institute of Cancer ResearchSutton, Surrey, United Kingdom; 5 Department of Internal Medicine I, Division of Hematology and Blood Coagulation, Medical University of ViennaVienna, Austria

## Abstract

The C allele of the rs2736100 single nucleotide polymorphism located in the second intron of the *TERT* gene has recently been identified as a susceptibility factor for myeloproliferative neoplasms (MPN) in the Icelandic population. Here, we evaluate the role of *TERT* rs2736100_C in sporadic and familial MPN in the context of the previously identified *JAK2* GGCC predisposition haplotype. We have confirmed the *TERT* rs2736100_C association in a large cohort of Italian sporadic MPN patients. The risk conferred by *TERT* rs2736100_C is present in all molecular and diagnostic MPN subtypes. *TERT* rs2736100_C and *JAK2* GGCC are independently predisposing to MPN and have an additive effect on disease risk, together explaining a large fraction of the population attributable fraction (PAF = 73.06%). We found *TERT* rs2736100_C significantly enriched (*P* = 0.0090) in familial MPN compared to sporadic MPN, suggesting that low-penetrance variants may be responsible for a substantial part of familial clustering in MPN. Am. J. Hematol. 89:1107–1110, 2014. © 2014 The Authors. American Journal of Hematology published by Wiley Periodicals, Inc.

## Introduction

Myeloproliferative neoplasms (MPN) constitute a group of phenotypically diverse chronic myeloid malignancies including three major disease entities: polycythemia vera (PV), essential thrombocythemia (ET), and primary myelofibrosis (PMF). Mutually exclusive oncogenic somatic mutations in three genes have been identified in more than 90% of MPN cases 1. Mutations in *JAK2*, most notably *JAK2*-V617F are present in all three disease subtypes, more frequently in PV 2–5, while *MPL* and *CALR* mutations are exclusively found in ET and PMF patients 6–8. The same somatic mutations are present also in familial MPN, which account for 5–10% of MPN cases 9–11. Previously, a common haplotype (GGCC or 46/1) at the *JAK2* locus has been found to predispose to *JAK2* mutation positive sporadic and familial MPN 12–15. Genome-wide association studies have successfully revealed risk loci for a series of cancers 16. A recent study identified the germline sequence variant rs2736100_C located in the second intron of the *TERT* gene as risk variant for MPN in the Icelandic population 17. In this study, we were seeking to confirm the *TERT* association in an independent cohort from a different ethnic background and evaluate the role of the *TERT* predisposition locus in familial clustering of MPN. Furthermore, we were testing the possibility of an interaction of *TERT* and *JAK2* susceptibility loci in sporadic and familial MPN.

## Methods

Blood samples from sporadic MPN (*n* **=** 717), familial MPN (*n* **=** 121) and control (*n* **=** 202) subjects from Italy were obtained after written informed consent. The study was approved by the institutional ethics committee (Comitato di Bioetica, Fondazione IRCCS Policlinico San Matteo) and procedures were in accordance with the Helsinki declaration. Details on patient characteristics and sample collection have been described in a previous study 15. Patients were defined as familial cases if two or more individuals within the same pedigree were affected. For each family, the proband was identified as the first affected family member seeking medical attention.

Presence of the *JAK2*-V617F mutation, *JAK2* exon 12 mutations, *MPL* exon 10 mutations, and *CALR* exon 9 mutations was assessed in granulocyte DNA as previously described 7. Genotyping for rs2736100 (*TERT*) and rs10974944 (*JAK2*) was performed using commercially available TaqMan SNP genotyping assays (C___1844009 and C__31941696, respectively; Applied Biosystems, Foster City, CA).

Statistical analyses were performed using the R statistical software (version 3.0.3) 18 in conjunction with the R-packages “SNPassoc” and “scrime.” Population attributable fraction (PAF) and proportions of familial relative risk (FRR) explained by *TERT* and *JAK2* loci were calculated as previously described by others 19,20, using an estimate of 5.6 for the overall MPN FRR 21. The Cochran–Armitage test of trend was applied to study differences in distribution of risk allele numbers per individual in the different cohorts. The absolute risk for developing MPN in different *TERT*/*JAK2* genotypic classes was calculated using logistic regression.

## Results

We confirmed the previously reported 17 association of rs2736100_C with the MPN phenotype in a cohort of sporadic MPN patients (*n* **=** 717) of a different ethnic background (Table1). The size of our sporadic MPN cohort allowed for comparison of genotype distributions in different molecular and diagnostic subgroups. Significant associations were detected for both *JAK2*-positive and *CALR*-positive MPN at similar strength (Table1), suggesting that there is no preferential susceptibility to any molecular subtype. A similar trend was observed for *MPL*-positive and triple negative MPN patients (lacking *JAK2*, *MPL*, and *CALR* mutations), however, statistical significance is absent, possibly due to low sample sizes (Supporting Information Table 1). As in the prior study 17, the *TERT* rs2736100_C association was present in PV, ET, and PMF, implying a general role in MPN pathogenesis (Supporting Information Table 1).

**Table 1:** Association of *TERT* rs2736100 with Sporadic and Familial MPN and Molecular Subtypes

		Genotype frequency (%) case population			Genotype frequency (%) control population			Odds ratio (95% CI)			
Case population	Control population	A/A	A/C	C/C	A/A	A/C	C/C	A/A	A/C	C/C	*P* value
Sporadic MPN(*n* = 717)	Control(*n* = 202)	11.3(81)	46.2 (331)	42.5 (305)	23.3 (47)	43.6 (88)	33.2 (67)	1	2.18 (1.42–3.35)	2.64 (1.69–4.13)	1.15 × 10^−4^
Sporadic MPN JAK2+ (*n* = 516)	Control (*n* = 202)	10.7(55)	44.8 (231)	44.6 (230)	23.3 (47)	43.6 (88)	33.2 (67)	1	2.24 (1.42–3.55)	2.93 (1.82–4.72)	5.55 × 10^−5^
Sporadic MPN CALR+ (*n* = 126)	Control (*n* = 202)	11.9 (15)	46.8 (59)	41.3 (52)	23.3 (47)	43.6 (88)	33.2 (67)	1	2.10 (1.08–4.10)	2.43 (1.23–4.82)	0.0270
Familial MPN (*n* = 121)	Control (*n* = 202)	5.0 (6)	39.7 (48)	55.4 (67)	23.3 (47)	43.6 (88)	33.2 (67)	1	4.27 (1.7–10.72)	7.83 (3.14–19.55)	1.10 × 10^−6^
Familial MPN probands (*n* = 75)	Control (*n* = 202)	5.3 (4)	36.0 (27)	58.7 (44)	23.3 (47)	43.6 (88)	33.2 (67)	1	3.61 (1.19–10.92)	7.72 (2.60–22.94)	2.65 × 10^−5^
Familial MPN (*n* = 121)	Sporadic MPN (*n* = 717)	5.0 (6)	39.7 (48)	55.4 (67)	11.3 (81)	46.2 (331)	42.5 (305)	1	1.96 (0.81–4.73)	2.97 (1.24–7.08)	0.0090
Familial MPN probands (*n* = 75)	Sporadic MPN (*n* = 717)	5.3 (4)	36.0 (27)	58.7 (44)	11.3 (81)	46.2 (331)	42.5 (305)	1	1.65 (0.56–4.85)	2.92 (1.02–8.37)	0.0180

**Figure 1:**
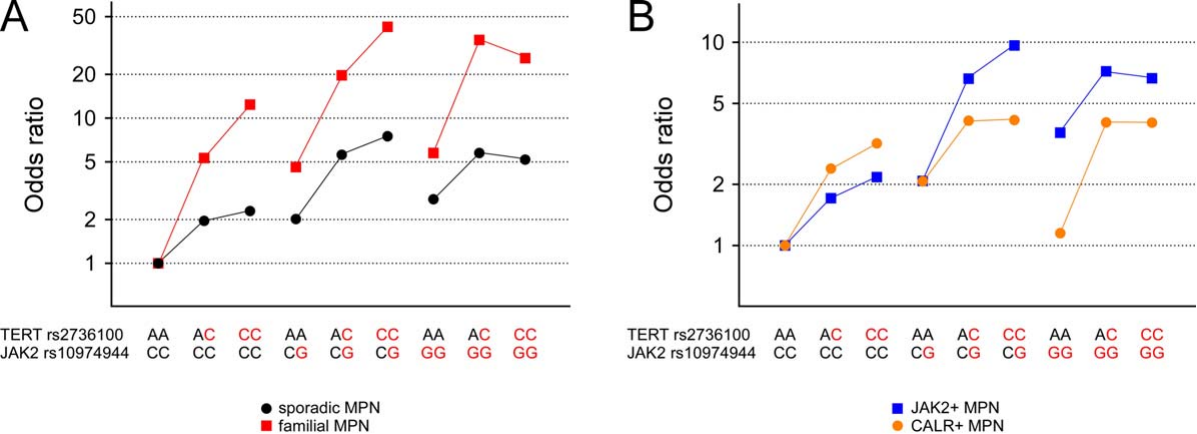
Combined effects of *TERT* and *JAK2* MPN predisposition loci. Genotypic odds ratios for MPN in respect to the nine genotypic combinations at *TERT* (rs2736100) and *JAK2* (rs10974944) loci are shown for (A) sporadic and familial total cohorts as well as for (B) sporadic *JAK2*-positive and *CALR*-positive subcohorts. Risk alleles are marked in red. [Color figure can be viewed in the online issue, which is available at wileyonlinelibrary.com.]

We next studied the combined effects of the two known MPN risk loci at *TERT* and *JAK2* as well as their possible interaction. Conditional logistic regression analysis revealed that *TERT* and *JAK2* loci are independently predisposing to MPN, the total combined risk approximating the sum of the two genotypes (Fig. 1; Supporting Information Table 2). Direct testing for interaction was negative (Supporting Information Table 3). The combined effects of *TERT* and *JAK2* risk loci are stronger in *JAK2*-positive MPN (Fig. 1). This finding is compatible with the *JAK2* risk haplotype predisposing primarily for the acquisition of somatic mutations in the *JAK2* gene.

To evaluate the role of *TERT* in familial MPN, we determined the rs2736100 genotype in 121 affected members of 75 Italian families with two or more MPN cases within first and/or second degree relatives. The *TERT* rs2736100_C association is significantly stronger in familial MPN compared to sporadic MPN (*P* **=** 0.009; Table1), indicating a role of the *TERT* locus in MPN familial clustering. Proband-based analysis confirmed this observation (*P* **=** 0.018; Table1). As previously reported on a smaller familial MPN cohort 15, such a significant difference in association strength is absent for *JAK2* GGCC when directly comparing sporadic and unfiltered familial *JAK2*-positive MPN cases (Supporting Information Table 4). However, MPN families consisting of both *JAK2*-positive and *JAK2*-negative members might be confounding the possible association, as *JAK2* GGCC predisposes mainly to *JAK2*-positive MPN. Indeed, when restricting analysis to families exclusively consisting of *JAK2*-positive members, we observed a significant trend toward *JAK2* GGCC enrichment in familial MPN compared to sporadic MPN (*P* **=** 0.046; Supporting Information Table 4). Moreover, the familial MPN cohort exhibits enrichment for individuals carrying more than three risk alleles (counting one for heterozygotes and two for homozygotes; Supporting Information Figure 1) and a trend for odds ratios increasing toward double positive (*TERT*/*JAK2*) individuals (Supporting Information Table 2), further supporting the possibility of *JAK2* GGCC contributing to MPN familial clustering. Independence of *TERT* and *JAK2* loci in mediating MPN susceptibility, as observed in sporadic MPN, is also true for familial MPN (Fig. 1).

Similar to that reported in the Icelandic study 17, in this study cohort *TERT* rs2736100_C is estimated to account for 51.89% of PAF. In concert with *JAK2* GGCC (PAF **=** 44.01%), they explain a large part (combined PAF **=** 73.06%) of the population susceptibility for MPN. The absolute risk for developing MPN in different *TERT*/*JAK2* genotypic classes is calculated in Supporting Information Table 5. As the risk variant rs2736100_C is present at high frequency (55%; Table1) in the general population, only 2.01% of the FRR can be attributed to the *TERT* rs2736100_C variation. In contrast, 5.15% of FRR can be explained by the *JAK2* GGCC risk haplotype which is present at lower frequency (27%; Supporting Information Table 4) in the general population.

## Discussion

The *TERT* gene encodes the reverse transcriptase of the telomerase complex, essential for maintaining telomere length 22. The C allele of rs2736100 has been linked to longer telomeres 23,24, compatible with a direct regulatory effect of rs2736100 genotype on *TERT* expression. *TERT* rs2736100_C was previously shown to also associate with elevated risk for several other cancers, albeit with lower effect 25–28. Furthermore, rs2736100_C is linked to increased blood cell count values 17,29, a hallmark of MPN. The fact that TERT rs2736100_C predisposes to all MPN subtypes implies a generic role in MPN predisposition, possibly through affecting blood cells counts.

Enrichment of common susceptibility loci in familial forms of predominantly sporadic cancers has been reported previously 20, and contribution of low-penetrance risk loci to familial clustering is well acknowledged 19,30. Notably, both *TERT* rs2736100_C for all MPN subtypes as well as *JAK2* GGCC for *JAK2*-positive MPN exhibit effect sizes stronger than typically observed for common cancer-predisposing variants 16. This might explain the significance of familial enrichment for *TERT* rs2736100_C (Table1). The implication of *JAK2* GGCC in familial clustering remains to be confirmed in a larger set of *JAK2*-positive MPN families.

In conclusion, common variation at *TERT* and *JAK2* loci explains most of the population risk for developing MPN. Enrichment of the *TERT* risk variant in familial MPN suggests the possibility of random accumulation of several common high frequency variants being responsible for parts of the elevated risk underlying familial clustering. More common low penetrance and/or higher penetrance rare mutations remain to be discovered to explain the missing heritability in sporadic MPN and the missing excess familial risk in MPN.
