# Supplementary material for: Common germline variation at the TERT locus contributes to familial clustering of myeloproliferative neoplasms
Source: Am J Hematol. 2014 Sep 26;89(12):1107–10. doi: 10.1002/ajh.23842 (PMC4657470; doi:10.1002/ajh.23842)
Supplement: Supplementary file 1 [file ajh0089-1107-sd1.doc]

**Supplementary Information**


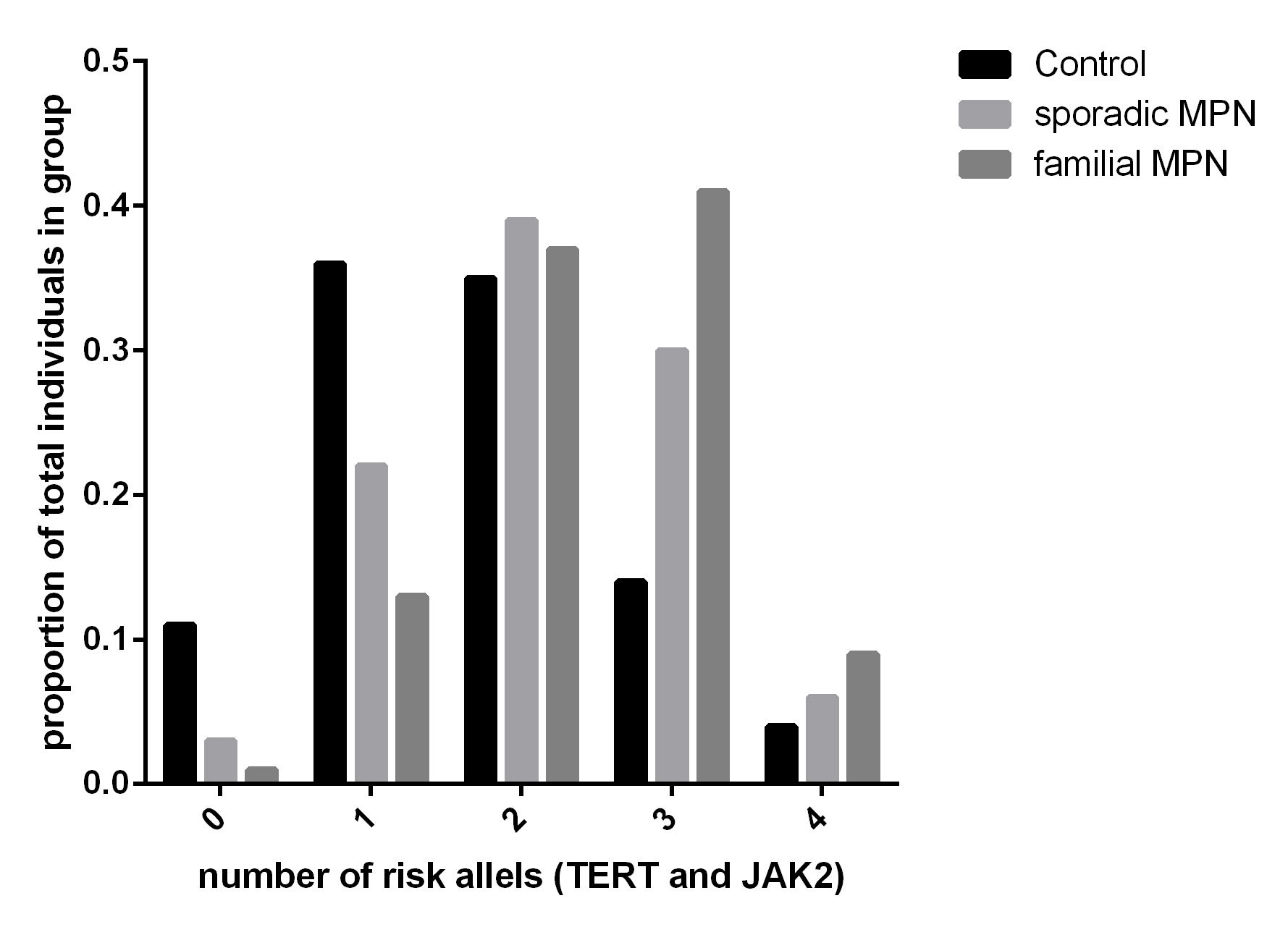


**Supplementary Figure 1: Relationship between number of risk alleles and sporadic/familial form of MPN.** The distribution of the number of risk alleles per individual for the control, sporadic MPN and familial MPN cohorts is shown. The Cochran-Armitage test of trend was applied to study differences in distribution of risk allele numbers, suggesting increased numbers of risk alleles in familial MPN compared to sporadic MPN cases, albeit not achieving formal statistical significance (*P_Trend_*=0.053).

**Supplementary Table 1:** *TERT* rs2736100 SNP association with MPN and its molecular and diagnostic subtypes (continuation of Table 1)

| Case population | Control population | Genotype frequency (%) case population | | | Genotype frequency (%) control population | | | Odds ratio (95% CI) | | | P value |
| --- | --- | --- | --- | --- | --- | --- | --- | --- | --- | --- | --- |
|  |  | A/A | A/C | C/C | A/A | A/C | C/C | A/A | A/C | C/C |  |
| Sporadic MPN MPL+ (n=25) | Control  (n=202) | 16.0  (4) | 52.0  (13) | 32.0  (8) | 23.3  (47) | 43.6  (88) | 33.2  (67) | 1 | 1.74 (0.54-5.62) | 1.40 (0.40-4.93) | 0.6301 |
| Sporadic MPN triple negative (n=50) | Control  (n=202) | 14.0  (7) | 56.0  (28) | 30.0  (15) | 23.3  (47) | 43.6  (88) | 33.2  (67) | 1 | 2.14 (0.87-5.26) | 1.50 (0.57-3.97) | 0.2033 |
| PV  (n=271) | Control  (n=202) | 8.9  (24) | 47.2  (128) | 43.9  (119) | 23.3  (47) | 43.6  (88) | 33.2  (67) | 1 | 2.85 (1.62-5.00) | 3.48 (1.96-6.19) | 5.36 x 10^-5^ |
| ET  (n=374) | Control  (n=202) | 13.6  (51) | 44.4  (166) | 42.0  (157) | 23.3  (47) | 43.6  (88) | 33.2  (67) | 1 | 1.74 (1.08-2.79) | 2.16 (1.32-3.52) | 8.41 x 10^-3^ |
| PMF  (n=72) | Control  (n=202) | 8.3  (6) | 51.4  (37) | 40.3  (29) | 23.3  (47) | 43.6  (88) | 33.2  (67) | 1 | 3.29 (1.30-8.37) | 3.39 (1.30-8.81) | 0.0129 |
| Familial MPN JAK2+  (n=88) | Control  (n=202) | 3.4  (3) | 42.0  (37) | 54.5  (48) | 23.3  (47) | 43.6  (88) | 33.2  (67) | 1 | 6.59 (1.93-22.5) | 11.22 (3.3-38.2) | 3.45 x 10^-6^ |
| Familial MPN CALR+  (n=12) | Control  (n=202) | 8.3  (1) | 33.3  (4) | 58.3  (7) | 23.3  (47) | 43.6  (88) | 33.2  (67) | 1 | 2.14 (0.23-19.7) | 4.91 (0.58-41.2) | 0.1737 |
| Familial MPN triple negative (n=21) | Control  (n=202) | 9.5  (2) | 33.3  (7) | 57.1  (12) | 23.3  (47) | 43.6  (88) | 33.2  (67) | 1 | 1.87 (0.37-9.36) | 4.21 (0.90-19.7) | 0.0747 |

**Supplementary Table 2:** Interaction of *TERT* and *JAK2* risk loci. Odds ratios for all possible *JAK2/TERT* genotype combinations are provided for sporadic MPN, familial MPN, JAK2+ sporadic MPN and CALR+ sporadic MPN cases.

| Sporadic MPN vs. control |  |  | TERT rs2736100 |  |  |
| --- | --- | --- | --- | --- | --- |
|  |  | A/A | A/C | C/C | P (interaction) |
|  | C/C | 1 | 1.96 (1.02-3.78) | 2.29 (1.16-4.5) | 0.84407 |
| JAK2 rs10974944 | C/G | 2.02 (0.93-4.39) | 5.59 (2.79-11.17) | 7.5 (3.6-15.6) |  |
|  | G/G | 2.76 (0.78-9.78) | 5.75 (2.25-14.67) | 5.18 (2.02-13.27) |  |
| Familial MPN vs. control |  |  | TERT rs2736100 |  |  |
|  |  | A/A | A/C | C/C | P (interaction) |
|  | C/C | 1 | 5.31 (0.65-43.25) | 12.38 (1.56-98.23) | 0.66795 |
| JAK2 rs10974944 | C/G | 4.6 (0.47-44.59) | 19.71 (2.48-156.96) | 42.55 (5.35-338.66) |  |
|  | G/G | 5.75 (0.3-111.8) | 34.5 (3.85-309) | 25.87 (2.82-237.5) |  |
| JAK2+ MPN vs. control |  |  | TERT rs2736100 |  |  |
|  |  | A/A | A/C | C/C | P (interaction) |
|  | C/C | 1 | 1.71 (0.82-3.58) | 2.17 (1.02-4.63) | 0.60728 |
| JAK2 rs10974944 | C/G | 2.08 (0.89-4.9) | 6.62 (3.1-14.13) | 9.63 (4.36-21.27) |  |
|  | G/G | 3.59 (0.96-13.5) | 7.19 (2.67-19.37) | 6.65 (2.46-17.99) |  |
| CALR+ MPN vs. control |  |  | TERT rs2736100 |  |  |
|  |  | A/A | A/C | C/C | P (interaction) |
|  | C/C | 1 | 2.39 (0.82-6.98) | 3.18 (1.08-9.42) | 0.95909 |
| JAK2 rs10974944 | C/G | 2.07 (0.6-7.2) | 4.11 (1.36-12.43) | 4.14 (1.3-13.18) |  |
|  | G/G | 1.15 (0.1-12.62) | 4.03 (0.99-16.35) | 4.02 (0.99-16.35) |  |

**Supplementary Table 3:** Logistic regression analysis of combined *TERT* and *JAK2* effect on MPN predisposition, without (top) and with (bottom) consideration of the interaction term.

|  | Estimate | SE | *P* value | |
| --- | --- | --- | --- | --- |
| (Intercept)* | -0.06106 | 0.21120 | 0.7725 |  |
| JAK2-C/G | 1.02512 | 0.17702 | **6.99 x 10^-9^** |  |
| JAK2-G/G | 0.96883 | 0.27200 | **3.68 x 10^-4^** |  |
| TERT-A/C | 0.83794 | 0.22575 | **2.06 x 10^-4^** |  |
| TERT-C/C | 1.01122 | 0.23411 | **1.56 x 10^-5^** |  |
| (Intercept) | 0.08338 | 0.28893 | 0.7729 |  |
| TERT-A/C | 0.67490 | 0.33424 | **0.0435** |  |
| TERT-C/C | 0.82777 | 0.34558 | **0.0166** |  |
| JAK2-C/G | 0.70508 | 0.39523 | 0.0744 |  |
| JAK2-G/G | 1.01523 | 0.64561 | 0.1158 |  |
| TERT-A/C:JAK2-C/G | 0.34023 | 0.47544 | 0.4742 |  |
| TERT-C/C:JAK2-C/G | 0.48179 | 0.49828 | 0.3336 |  |
| TERT-A/C:JAK2-G/G | 0.05906 | 0.76815 | 0.9387 |  |
| TERT-C/C:JAK2-G/G | -0.19916 | 0.77459 | 0.7971 |  |

*corrected intercept = -8.92827 (corrected using the real data for MPN prevalence in the population (5 x 10^-4^)); SE, standard error.

**Supplementary Table 4:** *JAK2* rs10974944 SNP association with MPN and its molecular and diagnostic subtypes

| Case population | Control population | Genotype frequency (%) case population | | | Genotype frequency (%) control population | | | Odds ratio (95% CI) | | | P value |
| --- | --- | --- | --- | --- | --- | --- | --- | --- | --- | --- | --- |
|  |  | C/C | C/G | G/G | C/C | C/G | G/G | C/C | C/G | G/G |  |
| Sporadic MPN  (n=717) | Control  (n=202) | 32.5  (233) | 52.6  (377) | 14.9  (107) | 56.4  (114) | 33.7  (68) | 9.9  (20) | 1 | 2.71 (1.93-3.82) | 2.62 (1.54-4.44) | 7.28 x 10^-9^ |
| Sporadic MPN JAK2+ (n=516) | Control  (n=202) | 26.6  (137) | 56.6  (292) | 16.9  (87) | 56.4  (114) | 33.7  (68) | 9.9  (20) | 1 | 3.57 (2.49-5.13) | 3.62 (2.10-6.25) | 9.18 x 10^-13^ |
| Sporadic MPN CALR+ (n=126) | Control  (n=202) | 46.8  (59) | 41.3  (52) | 11.9  (15) | 56.4  (114) | 33.7  (68) | 9.9  (20) | 1 | 1.48 (0.92-2.38) | 1.45 (0.69-3.04) | 0.2371 |
| Sporadic MPN MPL+ (n=25) | Control  (n=202) | 48.0  (12) | 52.0  (13) | 0.0  (0) | 56.4  (114) | 33.7  (68) | 9.9  (20) | 1 | 1.82 (0.78-4.21) | 0 | 0.0957 |
| Sporadic MPN triple negative (n=50) | Control  (n=202) | 50.0  (25) | 40.0  (20) | 10.0  (5) | 56.4  (114) | 33.7  (68) | 9.9  (20) | 1 | 1.34 (0.69-2.60) | 1.14 (0.39-3.33) | 0.6859 |
| PV  (n=271) | Control  (n=202) | 19.9  (54) | 59.0  (160) | 21.0  (57) | 56.4  (114) | 33.7  (68) | 9.9  (20) | 1 | 4.97 (3.23-7.64) | 6.02 (3.29-11.0) | 1.31 x 10^-15^ |
| ET  (n=374) | Control  (n=202) | 42.0  (157) | 46.8  (175) | 11.2  (42) | 56.4  (114) | 33.7  (68) | 9.9  (20) | 1 | 1.87 (1.29-2.70) | 1.52 (0.85-2.74) | 3.27 x 10^-3^ |
| PMF  (n=72) | Control  (n=202) | 30.6  (22) | 58.3  (42) | 11.1  (8) | 56.4  (114) | 33.7  (68) | 9.9  (20) | 1 | 3.2 (1.76-5.81) | 2.07 (0.81-5.30) | 4.44 x 10^-4^ |
| Familial MPN  (n=121) | Control  (n=202) | 28.1  (34) | 53.7  (65) | 18.2  (22) | 56.4  (114) | 33.7  (68) | 9.9  (20) | 1 | 3.21 (1.92-5.35) | 3.69 (1.80-7.55) | 3.25 x 10^-6^ |
| Familial MPN probands (n=75) | Control  (n=202) | 28.0  (21) | 54.7  (41) | 17.3  (13) | 56.4  (114) | 33.7  (68) | 9.9  (20) | 1 | 3.27 (1.79-6.00) | 3.53 (1.52-8.17) | 1.09 x 10^-4^ |
| Familial MPN JAK2+  (n=88) | Control  (n=202) | 20.5  (18) | 55.7  (49) | 23.9  (21) | 56.4  (114) | 33.7  (68) | 9.9  (20) | 1 | 4.56 (2.46-8.47) | 6.65 (3.02-14.6) | 2.62 x 10^-8^ |
| Familial MPN CALR+  (n=12) | Control  (n=202) | 50.0  (6) | 41.7  (5) | 8.3  (1) | 56.4  (114) | 33.7  (68) | 9.9  (20) | 1 | 1.40 (0.41-4.75) | 0.95 (0.11-8.32) | 0.8539 |
| Familial MPN triple negative (n=21) | Control  (n=202) | 47.6  (10) | 52.4  (11) | 0.0  (0) | 56.4  (114) | 33.7  (68) | 9.9  (20) | 1 | 1.84 (0.74-4.57) | 0 | 0.1224 |
| Familial MPN  (n=121) | Sporadic MPN  (n=717) | 28.1  (34) | 53.7  (65) | 18.2  (22) | 32.5  (233) | 52.6  (377) | 14.9  (107) | 1 | 1.18 (0.76-1.85) | 1.41 (0.79-2.52) | 0.5074 |
| Familial MPN probands (n=75) | Sporadic MPN  (n=717) | 28.0  (21) | 54.7  (41) | 17.3  (13) | 32.5  (233) | 52.6  (377) | 14.9  (107) | 1 | 1.21 (0.70-2.09) | 1.35 (0.65-2.79) | 0.6871 |
| Familial MPN JAK2+  (n=88) | Sporadic MPN JAK2+ (n=516) | 20.5  (18) | 55.7  (49) | 23.9  (21) | 26.6  (137) | 56.6  (292) | 16.9  (87) | 1 | 1.28 (0.72-2.27) | 1.84 (0.93-3.64) | 0.2158 |
| Familial MPN JAK2+ probands (n=55) | Sporadic MPN JAK2+ (n=516) | 16.4  (9) | 61.8  (34) | 21.8  (12) | 26.6  (137) | 56.6  (292) | 16.9  (87) | 1 | 1.77 (0.83-3.80) | 2.10 (0.85-5.19) | 0.2046 |
| Familial MPN all JAK2+  (n=60) | Sporadic MPN JAK2+ (n=516) | 18.3  (11) | 51.7  (31) | 30.0  (18) | 26.6  (137) | 56.6  (292) | 16.9  (87) | 1 | 1.32 (0.65-2.71) | 2.58 (1.16-5.72) | 0.0463 |

**Supplementary Table 5:** Absolute risk for developing MPN in different *TERT/JAK2* genotypic classes calculated using logistic regression.

|  |  | TERT rs2736100 | | |
| --- | --- | --- | --- | --- |
|  |  | A/A | A/C | C/C |
|  | C/C | 0.13 x 10^-3^ | 0.31 x 10^-3^ | 0.36 x 10^-3^ |
| JAK2 rs10974944 | C/G | 0.37 x 10^-3^ | 0.85 x 10^-3^ | 1.02 x 10^-3^ |
|  | G/G | 0.35 x 10^-3^ | 0.81 x 10^-3^ | 0.96 x 10^-3^ |
